# Associations Between Emotional Competence and Emotional Empathy in Secondary-Level Vocational Nursing Students at One Croatian School: A Cross-Sectional Study

**DOI:** 10.3390/ejihpe16080119

**Published:** 2026-08-18

**Authors:** Martina Trnčević, Ivana Pišćenec, Višnja Pranjić, Ljerka Armano, Aleksandar Racz

**Affiliations:** 1 Vinogradska Nursing School, Vinogradska Cesta 29, 10000 Zagreb, Croatia; martina.trncevic@skole.hr (M.T.); ivana.piscenec@skole.hr (I.P.); vpranjic@skolamedvinogradska.hr (V.P.); 2 Faculty of Medicine, University of Rijeka, Ul. Braće Branchetta 20, 51000 Rijeka, Croatia; aracz@zvu.hr; 3 University Hospital Centre Sestre Milosrdnice, Vinogradska Cesta 29, 10000 Zagreb, Croatia; 4 Faculty of Health Studies, University of Rijeka, Ul. Viktora Cara Emina 5, 51000 Rijeka, Croatia; 5 Faculty of Health Studies, University of Zagreb, Mlinarska Cesta 38, 10000 Zagreb, Croatia

**Keywords:** emotional empathy, affective empathy, emotional competence, emotional intelligence, nursing students, nursing education, vocational education, adolescence, emotion regulation, health professions education

## Abstract

Empathy is essential to humanized nursing care, yet its relationship with emotional competence before professional entry remains insufficiently specified. This single-school cross-sectional study examined whether distinct dimensions of emotional competence were differentially associated with emotional empathy among 337 secondary-level vocational nursing students enrolled in a five-year Croatian program for general care nurses/medical technicians. Students completed the Emotional Skills and Competence Questionnaire (ESCQ-45) and the Emotional Empathy Scale from the E-Questionnaire, which primarily captures affective responsiveness to others’ distress and other negative-valence states. Spearman correlations, Kruskal–Wallis tests with Dunn–Holm post hoc comparisons, Welch tests, and hierarchical linear regression with heteroscedasticity-consistent type 3 robust standard errors were used. Emotional empathy correlated with emotion perception and understanding (ρ = 0.35, *p* < 0.001) and emotion management (ρ = 0.28, *p* < 0.001), but not with emotion expression and naming (ρ = 0.10, *p* = 0.070). In the fully adjusted regression model, emotion perception and understanding (β = 0.254, *p* < 0.001), emotion management (β = 0.254, *p* = 0.003), and female versus male reported sex (β = 0.277, *p* < 0.001) were statistically significant predictors, whereas clinical exposure was not (*p* = 0.429). Emotion expression and naming had a negative adjusted coefficient (β = −0.158, *p* = 0.040) after simultaneous control for the remaining predictors. The findings support a dimensional interpretation of emotional competence and indicate that negative-valence emotional empathy is more closely associated with recognizing, understanding, and managing emotions than with expressive emotional skills considered in isolation.

## 1. Introduction

Empathy is a multidimensional capacity that involves understanding and affectively responding to another person’s emotional state while preserving the distinction between self and other (Davis, 1983; Håkansson Eklund & Meranius, 2021). Cognitive empathy concerns inferring or understanding another person’s perspective and emotions, whereas affective empathy concerns sharing or resonating with another person’s emotional experience. In healthcare, these processes contribute to therapeutic relationships, person-centered communication, humanized care, and professional practice (Moudatsou et al., 2020). Their development is especially relevant during adolescence, when empathic and prosocial responding remain sensitive to individual, relational, and educational influences (Silke et al., 2018).

Affective empathy is itself not valence-neutral. Contemporary models distinguish empathy for negative emotions, such as another person’s sadness, fear, pain, or distress, from positive empathy, defined as sharing and appreciating another person’s joy, relief, pride, or other positive affective states (Morelli et al., 2015; Brett et al., 2023). These components are related but not interchangeable and may show different associations with prosocial behavior, interpersonal closeness, well-being, and vulnerability to personal distress. The distinction is particularly important in nursing, where students and professionals encounter both suffering and positive clinical transitions. The Emotional Empathy Scale used in the present study predominantly assesses affective responsiveness and sympathetic reactions to people in distress. Accordingly, the present findings are interpreted primarily as evidence concerning negative-valence affective empathy; positive empathy was not directly measured.

Emotional competence refers to a set of skills involved in perceiving and understanding emotions, expressing and labeling emotional states, and managing and regulating emotions (Takšić et al., 2006). The ESCQ-45 operationalizes these capacities as three related dimensions. This framework is derived from the Mayer–Salovey model of emotional intelligence, in which accurate emotion perception supports emotional understanding, while emotion management represents a more integrative capacity for modulating emotional responses in oneself and in interpersonal situations (Mayer et al., 2004). Emotional competence may therefore support empathy through several mechanisms. Perception and understanding can improve recognition and interpretation of affective cues; emotion management can limit self-oriented personal distress and preserve attention to another person’s needs; and appropriately differentiated emotional expression can support communication after the emotional state has been recognized and regulated (Thompson et al., 2019; Zaki, 2020).

Empathy and emotional competence are conceptually connected but should not be treated as alternative labels for the same construct. Empathy is directed toward another person’s experience, whereas emotional competence includes broader intrapersonal and interpersonal skills for processing emotions. This distinction is relevant to the jangle fallacy, in which differently named constructs are assumed to be distinct without sufficient conceptual or empirical examination (Gonzalez et al., 2021). Conversely, empathy measures themselves often differ substantially in the processes and emotional valences they capture (Stosic et al., 2022). In the present study, the expected pattern was one of moderate association rather than redundancy: emotional competence dimensions were expected to explain part, but not all, of the variance in emotional empathy.

In health professions education, emotional intelligence and empathy have been associated with communication, interpersonal competence, and preparedness for clinical care (McNulty & Politis, 2023). However, comparison across nursing studies remains constrained by heterogeneous definitions, instruments, student populations, and educational designs (Jiao et al., 2022; Juniarta et al., 2024). Most available research concerns undergraduate students, whereas secondary-level vocational nursing students enter professional socialization during adolescence and encounter clinical demands before higher education or full professional practice. In Croatia, general care nurses and general care medical technicians are educated through a five-year secondary-level vocational program combining general education, vocational courses, and progressively increasing practical and clinical training (Ministry of Science, Education and Sports of the Republic of Croatia, 2013).

The present study examined the dimensional associations between emotional competence and emotional empathy among students at one Croatian vocational nursing school. Four hypotheses were specified. H1 proposed that emotion perception and understanding would be positively associated with emotional empathy. H2 proposed that emotion management would be positively associated with emotional empathy. H3 proposed that emotion expression and naming would show a weaker association with emotional empathy than the other two dimensions and would not make an independent positive contribution after their shared variance was controlled. H4 proposed that the associations of emotion perception and understanding and emotion management with emotional empathy would remain after adjustment for reported sex and clinical exposure at the time of assessment. Differences by year of education and reported sex were examined as contextual, exploratory analyses because previous findings have been inconsistent.

## 2. Materials and Methods

### 2.1. Study Design and Setting

This cross-sectional correlational study was conducted at one public vocational nursing school in Croatia. Data were collected between 3 October 2024 and 25 January 2025. Each participant completed the survey once; no intervention, experimental manipulation, or longitudinal follow-up was conducted.

### 2.2. Participants, Recruitment, and Sample Characteristics

The eligible population comprised students enrolled in the regular five-year program for general care nurses/general care medical technicians. A total of 337 students from the 1st, 3rd, 4th, and 5th years participated, representing 89.3% of students enrolled in those years. Inclusion criteria were enrollment in the program, sufficient comprehension of Croatian, and completion of the applicable consent and assent procedures. Students who had transferred from other secondary-level programs, completed primary education outside Croatia, or lacked the required consent were excluded to reduce heterogeneity in prior educational, language, and curricular background.

Second-year students were not included because vocational nursing education begins in the third year, while the second year remains within the general education phase of the curriculum. Because data collection occurred at the beginning of the school year, third-year students had not yet begun clinical training. Clinical exposure at the time of assessment was therefore coded as absent for 1st- and 3rd-year students and present for 4th- and 5th-year students. Participant characteristics are presented in Table 1.

### 2.3. Ethical Considerations

The study was conducted in accordance with the Declaration of Helsinki and was approved by the Ethics Committee of Vinogradska Nursing School (Ref. No. KL: 602-33/24-01/7; UR: 251-304-24-01; 18 April 2024). Written informed consent was obtained from parents or legal guardians for participants younger than 18 years, while participants aged 18 years or older provided their own written informed consent. All students received written study information and provided assent or consent before data collection. Participation was voluntary, and students could decline or discontinue without explanation, academic disadvantage, or loss of instructional time. Students who did not participate completed individual curriculum-based activities that extended the scheduled program content. No reimbursement, financial incentive, course credit, or other benefit was provided for participation.

### 2.4. Instruments and Measures

#### 2.4.1. Emotional Skills and Competence Questionnaire (ESCQ-45)

Emotional competence was assessed using the Emotional Skills and Competence Questionnaire (ESCQ-45), developed by Takšić and colleagues (Takšić et al., 2009). The ESCQ-45 comprises 45 items distributed across three subscales: Ability to Perceive and Understand Emotions (15 items), Ability to Express and Label Emotions (14 items), and Ability to Manage and Regulate Emotions (16 items). Items are rated from 1 (does not apply to me at all) to 5 (completely applies to me), with higher scores indicating greater self-reported emotional competence. Illustrative item content concerns recognizing another person’s emotional state from behavior, finding words to describe one’s feelings, and remaining able to manage emotions in demanding situations; these examples are paraphrased rather than reproduced verbatim.

To improve readability while preserving the original meaning and scoring, the subscales are referred to in the text and tables as emotion perception and understanding, emotion expression and naming, and emotion management. These shortened labels are semantic abbreviations of the published subscale names and do not represent newly defined constructs. Previous studies reported adequate internal consistency and cross-cultural invariance between Croatian and Portuguese versions (Costa et al., 2016; Takšić et al., 2009). Permission to use the instrument was obtained from the authors. In the present sample, Cronbach’s α was 0.90 for emotion perception and understanding, 0.86 for emotion expression and naming, and 0.81 for emotion management.

#### 2.4.2. Emotional Empathy Scale from the E-Questionnaire

Emotional empathy was assessed using the 19-item Emotional Empathy Scale from the E-Questionnaire developed by Raboteg-Šarić (2002). Items are rated from 0 (does not apply to me at all) to 4 (fully applies to me), producing a total score from 0 to 76; higher scores indicate greater emotional empathy. Illustrative content concerns feeling emotionally affected by another person’s suffering and experiencing sympathetic concern when another person is distressed. The scale therefore predominantly represents negative-valence affective empathy and sympathetic responsiveness rather than cognitive empathy or empathy for positive emotions. In the present sample, Cronbach’s α was 0.91.

### 2.5. Data Collection and Protection of Anonymity

Data were collected during regular class sessions through a Google Forms questionnaire under standardized conditions, using identical written instructions and the same time frame. The form was configured not to collect email addresses and could be accessed without signing in to a personal or institutional account. No names, contact details, account identifiers, internet protocol addresses available to the research team, or other direct personal identifiers were requested. The resulting dataset was anonymous at the point of collection rather than subsequently pseudonymized. The researcher was present only to provide technical or procedural clarification, had no role in grading the participating students, and did not influence responses. Completion time was approximately 25 min.

### 2.6. Statistical Analysis

Statistical analyses were performed using IBM SPSS Statistics version 26.0 (IBM Corp., Armonk, NY, USA) and independently verified from the original de-identified data using Python 3.13, SciPy 1.17, and statsmodels 0.14.6. Internal consistency was assessed using Cronbach’s α (Cronbach, 1951). Continuous variables were summarized using means, standard deviations, observed ranges, skewness, excess kurtosis, and, for rank-based group comparisons, medians and interquartile ranges. Normality of the four composite emotional scores was tested using the Shapiro–Wilk test (Shapiro & Wilk, 1965) and evaluated together with graphical diagnostics. Spearman rank correlations were used for bivariate associations (Spearman, 1904).

Because several bounded composite scores departed from normality, differences across years of education were evaluated using Kruskal–Wallis tests (Kruskal & Wallis, 1952), with epsilon-squared (ε^2^) as the effect-size estimate. When the omnibus test was statistically significant, Dunn pairwise comparisons were conducted using ranks from the combined sample (Dunn, 1964), and familywise error was controlled by the Holm step-down procedure (Holm, 1979). For the exploratory reported-sex analysis, the eight participants who selected the ‘not reported’ category were excluded, and male and female participants were compared using Welch independent-samples *t*-tests, which do not assume equal variances (Welch, 1947). Holm-adjusted *p*-values and Hedges’ g were reported for these four comparisons (Hedges, 1981).

Hierarchical multiple linear regression was used to estimate adjusted associations with emotional empathy as the outcome. Model 1 included the three ESCQ-45 dimensions. Model 2 additionally included reported sex (0 = male, 1 = female) and clinical exposure at assessment (0 = no clinical exposure [1st and 3rd years], 1 = clinical exposure [4th and 5th years]). Participants who did not report sex were excluded from both models to maintain a common complete-case sample (N = 329). Year of education was not entered simultaneously with clinical exposure because clinical exposure was deterministically derived from year within this curriculum. Linearity, residual form, heteroscedasticity, and influence were assessed using residual, quantile–quantile, scale–location, and Cook’s-distance diagnostics. Because the residual diagnostics indicated mild non-normality and heteroscedasticity, coefficient inference used heteroscedasticity-consistent type 3 (HC3) robust standard errors (Long & Ervin, 2000). Multicollinearity was evaluated using tolerance and the variance inflation factor (VIF); tolerance ≤ 0.20 or VIF ≥ 5 was used as a conservative warning threshold, while recognizing that such cut-offs require contextual interpretation (O’Brien, 2007).

Potential common-method variance was examined at item level using Harman’s single-factor diagnostic: all 45 ESCQ-45 items and the 19 Emotional Empathy items were entered into an unrotated principal-components analysis, and the proportion of variance explained by the first component was inspected (Harman, 1976; Podsakoff et al., 2003). This diagnostic was treated as a limited sensitivity check rather than proof that common-method bias was absent (Podsakoff et al., 2024). All tests were two-tailed. For primary analyses, *p* < 0.05 was considered statistically significant; adjusted *p*-values are explicitly identified where multiple-comparison correction was applied.

## 3. Results

### 3.1. Descriptive Statistics and Distributional Assessment

Descriptive and distributional statistics are shown in Table 2. Shapiro–Wilk testing indicated no statistically significant departure from normality for emotion expression and naming (W = 0.993, *p* = 0.105), whereas emotion perception and understanding (W = 0.978, *p* < 0.001), emotion management (W = 0.984, *p* = 0.001), and emotional empathy (W = 0.967, *p* < 0.001) departed from normality. The observed skewness values ranged from −0.68 to −0.05 and excess kurtosis values from 0.02 to 1.20. These results, considered together with the bounded score ranges and graphical diagnostics, supported rank-based methods for unadjusted correlations and year-group comparisons; regression assumptions and inference were evaluated separately.

### 3.2. Associations Between Study Variables

The three emotional competence dimensions were positively intercorrelated, with the strongest association between emotion expression and naming and emotion management (ρ = 0.61, *p* < 0.001). Emotional empathy correlated with emotion perception and understanding (ρ = 0.35, *p* < 0.001) and emotion management (ρ = 0.28, *p* < 0.001), but not with emotion expression and naming (ρ = 0.10, *p* = 0.070) (Table 3). The corresponding shared variance with emotional empathy was approximately 12%, 8%, and 1%, respectively, indicating related but non-redundant constructs.

### 3.3. Group Differences by Year of Education

The only statistically significant omnibus difference across years was found for emotion expression and naming, with H(3) = 9.52, *p* = 0.023, and ε^2^ = 0.02, indicating a small effect (Table 4). Dunn–Holm post hoc comparisons showed that fourth-year students had higher scores than first-year students (z = 2.90, adjusted *p* = 0.023). No other pairwise comparison was statistically significant after correction (adjusted *p* = 0.112–0.763). Emotion perception and understanding, emotion management, and emotional empathy did not differ significantly by year (Table 5).

### 3.4. Group Differences Between Male and Female Participants

The eight participants who did not report sex were excluded from the inferential comparison. Female participants reported higher emotional empathy than male participants, with M = 57.84 (SD = 11.07) versus M = 49.85 (SD = 10.62), Welch t(108.06) = 5.48, Holm-adjusted *p* < 0.001, and Hedges’ g = 0.73 (Table 6). No Holm-adjusted difference was found for emotion perception and understanding (adjusted *p* = 0.938), emotion expression and naming (adjusted *p* = 0.134), or emotion management (adjusted *p* = 0.627). The unadjusted expression-and-naming comparison was *p* = 0.045 but this did not remain statistically significant after correction.

### 3.5. Common-Method Diagnostic

Harman’s single-factor diagnostic included all 64 item-level indicators. Fourteen components had eigenvalues greater than 1, and the first unrotated component accounted for 19.29% of the total variance. Thus, the data did not yield a single dominant factor and the first component was well below the conventional 50% screening value. This result reduces, but does not eliminate, concern about a dominant common-method factor; the diagnostic cannot exclude more complex forms of method bias.

### 3.6. Hierarchical Regression Analysis

Model 1 explained 14.0% of the variance in emotional empathy, with R^2^ = 0.140, HC3 robust F(3, 325) = 10.25, and *p* < 0.001 (Table 7). Emotion perception and understanding (β = 0.277, *p* < 0.001) and emotion management (β = 0.249, *p* = 0.004) had positive adjusted coefficients. Emotion expression and naming had a negative adjusted standardized coefficient (β = −0.203, *p* = 0.005) after simultaneous control for the other emotional competence dimensions.

After reported sex and clinical exposure were added, Model 2 explained 21.8% of the variance, with R^2^ = 0.218, ΔR^2^ = 0.078, HC3 robust F(5, 323) = 16.12, and *p* < 0.001. Emotion perception and understanding (β = 0.254, *p* < 0.001), emotion management (β = 0.254, *p* = 0.003), and female versus male reported sex (β = 0.277, *p* < 0.001) were statistically significant predictors. Emotion expression and naming retained a negative adjusted coefficient (β = −0.158, *p* = 0.040), whereas clinical exposure was not statistically significant (β = −0.042, *p* = 0.429). The Model 2 residual Shapiro–Wilk test was statistically significant (W = 0.971, *p* < 0.001), but no highly influential case was detected (maximum Cook’s D = 0.167); HC3 robust inference was therefore used. H1, H2, H3, and H4 were supported within the operational definitions used in this study (Table 8).

## 4. Discussion

This study examined how three dimensions of emotional competence were associated with emotional empathy among secondary-level vocational nursing students at one Croatian school. Emotion perception and understanding and emotion management were positively associated with emotional empathy in both bivariate and adjusted analyses. Emotion expression and naming showed a weak, nonsignificant bivariate association and a negative adjusted standardized coefficient after the remaining predictors were controlled. Female versus male reported sex was statistically significant in the fully adjusted model, whereas the broad indicator of clinical exposure was not. These conclusions were unchanged when inference was based on HC3 robust standard errors.

The contribution of the study lies less in demonstrating a general relationship between emotional intelligence and empathy than in identifying a differentiated pattern before professional entry. Secondary-level vocational students represent an understudied population positioned between adolescent socio-emotional development and early clinical professionalization. The results indicate that emotional competence should not be treated as a single interchangeable resource: skills for recognizing, understanding, and managing emotions were more closely connected with the measured form of affective empathy than were expressive and naming skills considered in isolation.

### 4.1. Affective Empathy for Negative and Positive Emotions

The present measure primarily captured emotional responses to another person’s distress. This is important because negative and positive empathy are distinguishable components of affective empathy (Morelli et al., 2015; Brett et al., 2023). Empathy for distress may recruit emotion regulation to prevent vicarious arousal from becoming self-focused personal distress, whereas positive empathy may depend more strongly on the capacity to share, sustain, and celebrate another person’s positive state. The present results should therefore not be generalized to empathy for joy, relief, recovery, or achievement. In nursing education, both valences matter: students must respond constructively to pain, fear, and loss, but also recognize positive transitions such as recovery, successful adaptation, or regained autonomy.

### 4.2. Psychological Mechanisms Linking Emotional Competence and Empathy

The association between emotion perception and understanding and emotional empathy is consistent with a cue-processing and self–other differentiation mechanism. Empathic responding requires access to another person’s emotional signals while maintaining awareness that the observed state belongs to the other person rather than the self (Decety & Lamm, 2006; Lamm et al., 2011). Students who more readily identify facial, verbal, and situational indicators of emotion may construct a more accurate representation of another person’s affective state and may be better able to distinguish empathic resonance from undifferentiated emotional contagion. Contemporary integrative accounts likewise position emotion recognition, self–other distinction, and regulatory control as interacting rather than isolated processes (Thompson et al., 2019; Zaki, 2020).

Emotion management may support empathy through regulatory control. Vicarious distress can narrow attention toward one’s own discomfort, avoidance, or urgency to escape the situation; regulation can preserve attentional resources and keep an other-oriented response available. This interpretation is consistent with evidence distinguishing empathy from sympathy and personal distress and showing that the relation between empathy and regulation depends on the specific component measured (Yavuz et al., 2024). In nursing, regulation does not imply emotional detachment. Rather, it may allow emotional responsiveness to be sustained without overwhelming the student, confusing self and other, or displacing the patient’s needs (Decety & Lamm, 2006; Thompson et al., 2019; Zaki, 2020).

The pattern for emotion expression and naming requires a different explanation. This dimension was strongly associated with emotion management but was not bivariately associated with emotional empathy. Its negative adjusted coefficient emerged only after the variance shared with perception and management was controlled. This configuration is compatible with a suppression or net-effect phenomenon in multiple regression and should not be interpreted as evidence that emotional expression reduces empathy. The coefficient describes the association of the residualized portion of expression and naming at fixed levels of the other predictors; it may reflect self-focused expressive salience, verbal confidence, response style, or variance unrelated to other-oriented resonance. Replication with measures that separately assess alexithymia, expressive style, social desirability, personal distress, and positive and negative empathy is needed before a specific mechanism can be established.

### 4.3. Construct Distinctiveness and the Jangle-Fallacy Concern

The conceptual proximity of emotional competence and empathy raises a legitimate question about construct overlap. In this sample, however, their empirical associations were modest rather than redundant. Emotion perception and understanding shared approximately 12% of its variance with emotional empathy, emotion management approximately 8%, and emotion expression and naming approximately 1%. The measures also differ in referent and content: the ESCQ-45 assesses broad self-perceived skills for processing one’s own and others’ emotions, whereas the Emotional Empathy Scale assesses affective and sympathetic responsiveness to another person’s distress. Harman’s diagnostic did not identify a dominant single factor: the first unrotated component accounted for 19.29% of item variance. This finding argues against a single general response factor as the sole explanation, but it does not rule out more complex common-method effects, which require procedural controls and multimethod designs (Gonzalez et al., 2021; Podsakoff et al., 2003, 2024; Stosic et al., 2022).

### 4.4. Year of Education and Clinical Exposure

The omnibus year-of-education comparison was statistically significant only for emotion expression and naming, and the effect was small. Formal Dunn–Holm post hoc testing located the difference between first- and fourth-year students; no other pairwise comparison was statistically significant. Emotional empathy, emotion perception and understanding, and emotion management did not show significant omnibus differences. Because the study was cross-sectional and the pairwise pattern was not monotonic across years, the result cannot be interpreted as developmental growth or a causal effect of education. The broad clinical-exposure indicator was likewise not associated with emotional empathy in the regression model. It distinguished students only according to whether clinical education had begun and did not capture placement hours, patient populations, supervision quality, reflective learning, emotional demands, or clinical climate.

Previous nursing studies have reported inconsistent empathy patterns across educational stages, while longitudinal and intervention research suggests that change is not uniform across emotional dimensions or measurement approaches (Budler et al., 2022; Foster et al., 2017; Levett-Jones et al., 2019). Mere progression through a curriculum may therefore be insufficient. Educational experiences may influence empathy when they include structured reflection, simulation, debriefing, communication practice, and explicit integration of emotional learning. This interpretation is consistent with evidence supporting simulation-based and structured empathy interventions in nursing students (Cho & Kim, 2024; Ko et al., 2025).

### 4.5. Differences by Reported Sex

After exclusion of the ‘not reported’ category and correction across four comparisons, female participants reported higher emotional empathy than male participants, with a moderate-to-large standardized difference. No adjusted difference was detected for the three emotional competence dimensions. The expression-and-naming comparison reached *p* = 0.045 before correction but not after Holm adjustment and therefore was not interpreted as statistically significant. These results should remain cautious because the sample contained substantially more female than male students, the measures were self-reports, and the study did not assess gender identity, gender-role orientation, or social desirability. The findings describe score differences in this sample and cannot distinguish biological, socialization, selection, or measurement explanations.

### 4.6. Implications for Nursing Education and Clinical Relevance

The findings support a targeted rather than global approach to socio-emotional education. Learning activities may be most useful when they separately train recognition of affective cues, interpretation of emotional context, self–other differentiation, regulation of personal distress, and communication of an empathic response. Guided reflection, patient narratives, role-play, standardized-patient encounters, and simulation with debriefing can make these processes observable and discussable. Educational assessment should likewise avoid treating a single global emotional-intelligence score as a complete indicator of empathic readiness.

Clinical relevance can be strengthened by examining emotionally demanding and diagnostically specific situations. Future studies should compare responses to patients with dementia, severe mental illness, chronic pain, cancer, communication impairment, substance-use disorders, aggressive behavior, or end-of-life needs, because these contexts place different demands on cue recognition, regulation, perspective-taking, and empathic communication. Such work would connect the present dimensional findings more directly to difficult care situations and clinically meaningful performance.

### 4.7. Study Limitations

The study has several limitations. Its cross-sectional design precludes causal and developmental inference. The single-school sample limits transferability to other Croatian and international curricula, although participation covered 89.3% of eligible students in the included years. The pronounced imbalance in reported sex limits interpretation of group differences. All principal variables were collected by self-report in one survey session, creating possible social-desirability, response-style, and common-method effects. Although Harman’s first component accounted for only 19.29% of variance, this diagnostic is insensitive to some multifactorial forms of method bias and cannot establish their absence. The Emotional Empathy Scale predominantly captures negative-valence affective responsiveness and does not separately assess cognitive empathy, positive empathy, empathic concern, personal distress, or observable empathic behavior. Clinical exposure was a broad curriculum-derived indicator and did not measure dose or quality. Regression residuals showed mild non-normality and heteroscedasticity; HC3 robust standard errors were used to reduce sensitivity of inference to these departures. Finally, because the ESCQ-45 dimensions were intercorrelated, the negative adjusted coefficient for emotion expression and naming should be interpreted as a conditional coefficient within the multivariable model rather than a direct adverse relationship.

### 4.8. Future Research

Future research should use multicenter longitudinal designs beginning before clinical education and repeating assessment after clearly characterized placements. Positive and negative empathy should be measured separately, alongside cognitive and affective components, personal distress, social desirability, and emotion-regulation strategies. Ability-based tasks, behavioral observation, teacher or mentor ratings, and standardized-patient assessments would reduce reliance on a single method. Intervention studies should test whether training in emotion recognition, self–other differentiation, regulation, and reflective communication produces change in empathy and clinical performance and whether effects differ across patient diagnoses and emotionally demanding care scenarios. Measurement models should directly evaluate discriminant validity among emotional competence, empathy, sympathy, and related constructs.

## 5. Conclusions

Among secondary-level vocational nursing students at one Croatian school, emotional competence dimensions were not equally associated with emotional empathy. Emotion perception and understanding and emotion management showed consistent positive associations, while emotion expression and naming was weakly related at the bivariate level and had a negative adjusted coefficient only after shared variance and covariates were controlled. The findings apply primarily to negative-valence affective empathy because positive empathy and cognitive empathy were not measured. Within the limits of a single-site cross-sectional self-report design, the results support educational approaches that distinguish specific socio-emotional processes and connect them to clinically demanding interpersonal situations rather than treating emotional competence as one global attribute.

## Figures and Tables

**Table 1:** Participant characteristics (N = 337).

Characteristic	Category/Statistic	n	%
Age, years	Mean (SD)	17.28 (1.52)	—
Year of education	1st year	81	24.0
	3rd year	99	29.4
	4th year	68	20.2
	5th year	89	26.4
Reported sex	Male	68	20.2
	Female	261	77.4
	Not reported	8	2.4
Clinical exposure at assessment	No (1st and 3rd years)	180	53.4
	Yes (4th and 5th years)	157	46.6

Percentages may not total 100 because of rounding. Sex refers to the response category recorded in the study questionnaire; no gender-identity variable was collected.

**Table 2:** Descriptive statistics, distributional indices, normality tests, and internal consistency of the study measures.

Variable	N	Mean	SD	Observed Range	Skewness	Kurtosis	W	*p*	Cronbach’s α
Emotion perception and understanding	337	56.89	8.82	16–75	−0.41	1.20	0.978	<0.001	0.90
Emotion expression and naming	337	47.53	8.79	22–69	−0.05	0.02	0.993	0.105	0.86
Emotion management	337	60.12	7.84	29–80	−0.45	0.76	0.984	0.001	0.81
Emotional empathy	337	55.84	11.67	11–76	−0.68	0.89	0.967	<0.001	0.91

**Table 3:** Spearman correlations between emotional competence dimensions and emotional empathy (N = 337).

Variable	1	2	3	4
1. Emotion perception and understanding	—	0.37 (*p* < 0.001)	0.43 (*p* < 0.001)	0.35 (*p* < 0.001)
2. Emotion expression and naming		—	0.61 (*p* < 0.001)	0.10 (*p* = 0.070)
3. Emotion management			—	0.28 (*p* < 0.001)
4. Emotional empathy				—

Values are Spearman’s rank correlation coefficients (ρ) with two-tailed *p*-values. The upper triangle is presented.

**Table 4:** Differences by year of education.

Variable	1st Year (n = 81)	3rd Year (n = 99)	4th Year (n = 68)	5th Year (n = 89)	H(3)	ε^2^	*p*
Emotion perception and understanding	56 (49–63)	57 (53–62)	57 (52–61.5)	58 (51–63)	1.36	0.00	0.715
Emotion expression and naming	45 (39–50)	47 (42–53)	49 (43.5–54)	48 (43–53)	9.52	0.02	0.023
Emotion management	59 (54–65)	60 (56–65)	61.5 (56–66.5)	61 (57–67)	5.34	0.01	0.148
Emotional empathy	57 (50–67)	56 (50–63)	54 (44.5–64)	57 (49–64)	1.76	0.00	0.623

Scores are median (interquartile range). H = Kruskal–Wallis statistic; ε^2^ = epsilon-squared effect size. Omnibus and post hoc *p*-values are two-tailed.

**Table 5:** Dunn post hoc comparisons for emotion expression and naming by year of education.

Year 1	Year 2	z	Unadjusted *p*	Holm-Adjusted *p*
1st	3rd	−1.49	0.137	0.431
1st	4th	−2.90	0.004	0.023
1st	5th	−2.28	0.022	0.112
3rd	4th	−1.61	0.108	0.431
3rd	5th	−0.88	0.381	0.763
4th	5th	0.78	0.436	0.763

Dunn tests followed the statistically significant Kruskal–Wallis omnibus test. The sign of z reflects Year 1 minus Year 2 mean ranks. Holm-adjusted *p*-values control the familywise error rate across six pairwise comparisons.

**Table 6:** Welch independent-samples comparisons between male and female participants.

Variable	Male M (SD) (n = 68)	Female M (SD) (n = 261)	Welch t (df)	*p*	Holm-Adjusted *p*	Hedges’ g
Emotion perception and understanding	56.87 (9.73)	56.97 (8.62)	0.08 (96.14)	0.938	0.938	0.01
Emotion expression and naming	49.68 (9.83)	47.03 (8.47)	−2.03 (94.49)	0.045	0.134	−0.30
Emotion management	61.28 (9.37)	60.04 (7.34)	−1.01 (89.53)	0.313	0.627	−0.16
Emotional empathy	49.85 (10.62)	57.84 (11.07)	5.48 (108.06)	<0.001	<0.001	0.73

Positive Hedges’ g values indicate higher female scores; negative values indicate higher male scores. The eight participants who did not report sex were excluded. Holm adjustment was applied across the four outcome comparisons.

**Table 7:** Hierarchical regression models for emotional empathy using HC3 robust standard errors (complete-case N = 329).

Predictor/Statistic	Model 1 β	t	*p*	Model 2 β	t	*p*
Emotion perception and understanding	0.277	3.988	<0.001	0.254	3.732	<0.001
Emotion expression and naming	−0.203	−2.812	0.005	−0.158	−2.067	0.040
Emotion management	0.249	2.885	0.004	0.254	2.976	0.003
Reported sex	—	—	—	0.277	5.983	<0.001
Clinical exposure at assessment	—	—	—	−0.042	−0.793	0.429
R	0.374			0.467		
R^2^	0.140			0.218		
ΔR^2^	—			0.078		
Robust F (df1, df2)	F(3, 325) = 10.25			F(5, 323) = 16.12		
Model *p*	<0.001			<0.001		

Standardized regression coefficients (β) are reported. t and *p* values use heteroscedasticity-consistent type 3 (HC3) robust standard errors. R^2^ and ΔR^2^ are conventional ordinary-least-squares fit statistics. Model 1 included the three ESCQ-45 dimensions. Model 2 additionally included reported sex (0 = male, 1 = female) and clinical exposure at assessment (0 = no clinical exposure [1st and 3rd years], 1 = clinical exposure [4th and 5th years]).

**Table 8:** Collinearity diagnostics for the hierarchical regression models.

Predictor	Model 1 Tolerance	Model 1 VIF	Model 2 Tolerance	Model 2 VIF
Emotion perception and understanding	0.736	1.359	0.729	1.372
Emotion expression and naming	0.600	1.668	0.585	1.709
Emotion management	0.537	1.861	0.536	1.866
Reported sex	—	—	0.981	1.020
Clinical exposure at assessment	—	—	0.970	1.031

VIF = variance inflation factor. Conservative warning thresholds were tolerance ≤ 0.20 or VIF ≥ 5. All observed values were comfortably within the prespecified limits (Table 8).

## Data Availability

The data presented in this study are available on request from the corresponding author due to privacy and ethical restrictions concerning minors and educational data.
